# Comparison between camcorder, frontal head and temporal mounted action-cam in digestive surgery: Documentation and educational alternative during COVID-19 pandemic

**DOI:** 10.1016/j.amsu.2021.01.046

**Published:** 2021-01-23

**Authors:** Adeodatus Yuda Handaya, Aditya Rifqi Fauzi, Joshua Andrew, Ahmad Shafa Hanif, Azriel Farrel Kresna Aditya

**Affiliations:** a Digestive Surgery Division, Department of Surgery, Faculty of Medicine, Universitas Gadjah Mada/Dr. Sardjito Hospital, Yogyakarta, 55281, Indonesia; b Faculty of Medicine, Universitas Gadjah Mada/Dr. Sardjito Hospital, Yogyakarta, 55281, Indonesia

**Keywords:** Surgical recording, Camcorder, Actioncam, Digestive surgery, COVID-19 pandemic, Intraoperative videography

## Abstract

**Objective:**

COVID-19 pandemic has made impact both in clinical and educational settings. The number of surgeries has decreased; thus, the surgery videos of all cases are important for both documentation and education. This study aimed to compare three kinds of cameras in recording digestive surgery.

**Methods:**

We compared three cameras: Panasonic HV-770 Full HD Camcorder, Sony FDR-X3000 Action-cam, and Ordro EP7 Hands-Free FPV Camera. Each camera was used in several recording for superficial and visceral digestive surgeries and we compared the following: operation field, image focus, surgeon's comfort, practicality, and record settings.

**Results:**

Camcorder needs 10–15 min to set up and longer dismounting time, has steady vantage view and focus, good image quality, can be zoomed, but the recording may be obstructed by the surgeon's head. Action camera needs 5–10 min to set up and the dismounting time was equal between Camcorder and Ordro. Action camera depicts surgeon's vision, however, zoom could not be applied while recording. Sony FDR-X3000 used in this study had good image quality, but the use of this camera in a long surgery may generate neck stiffness due to its weight. Ordro EP7 was comfortable in any surgery but it had inferior image quality compared to the others.

**Conclusions:**

Panasonic HC-V770 and Sony FDR X3000 had good image quality, where camcorder excelled in longer surgeries due to its comfort, action-cam excelled for shorter surgeries due to ease of use and settings. Ordro EP7 was the most comfortable among all but has lowest image quality.

## Highlights

• COVID-19 pandemic has made impact both in clinical and educational settings.
• Surgery videos are important for both documentation and education in COVID-19 pandemic.
• We were comparing three cameras in recording digestive surgery to determine the best camera types in various settings.

## Abbreviations

FHD: Full high-definition
HMD: head mounted display

## 1 Introduction

Surgical recording is widely increasing in this era. Recording surgical procedures has numerous advantages. In clinical settings, surgery recording may give the ability to review the surgery process and results, aid in future management planning and referral, act as objective evidence if needed, serve audit purposes, and aid in the implementation of good medical practice. For surgeons, the ability to record procedures from their point of view offers opportunities to analyze surgical performance and provide objective feedback for appraisal and assessment [1]. In education settings, surgical procedure recording can help students to understand text-based materials and actual three-dimensional surgical field of view, demonstrating surgical procedures, assessing surgeons' and students’ skills to improve service quality, providing unique opportunity to see rare surgical cases, and act as research tools and visual supplements to medical journals. For patients, surgical videos may help the understanding of their body and condition [[1], [2], [3], [4], [5], [6], [7]].

The Coronavirus Disease 2019 (COVID-19) pandemic settings since 2020 have required health protocol, physical distancing, and even total lock down period all over the world and in all sectors, including education, and as a result, surgical recording can aid in shifting from conventional learning process to online teaching [5,8].

In surgical recordings, the camera type is a major factor to consider. Recording cameras are widely available in the market, and the one most used in surgery is the camera built into a head lamp. This camera has good video quality but is not suitable for all head lamp types, expensive, difficult to install, limiting surgeons’ movement, and needing monitoring in recording. The use of other types of cameras such as action camera with or without modifications have been reported recently. Many surgeons choose Full High Definition (FHD) cameras with wireless remote to monitor vantage points. In this study, we compared three cameras: one camcorder and two action-cam, with three different record position that are widely used in surgical recording to determine the best camera types and ways for surgery recording in various settings.

## 2 Materials and methods

The inclusion criteria of this study were: the patients agreed by signed informed consent to undergo digestive surgery recording in elective surgery cases, and the patients were confirmed COVID-19 free from screening and examinations. We exclude patients with emergency surgical cases. All equipment used in this study were disinfected with 70% alcohol prior to entering the operating room.

**Fig. 1:**
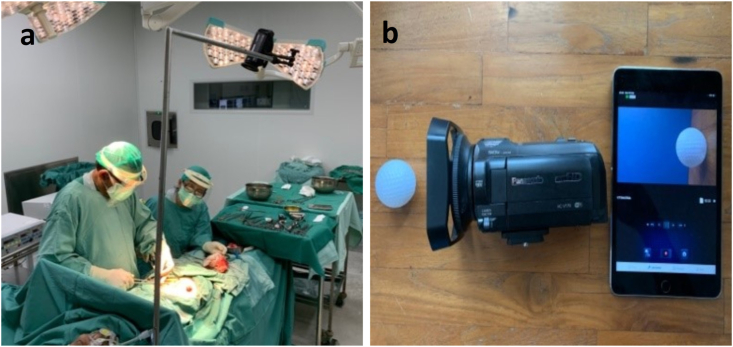
a) Physical appearance of the Panasonic HC-V770 when mounted; b) Physical appearance of the Panasonic HC-V770 when unmounted.

**Fig. 2:**
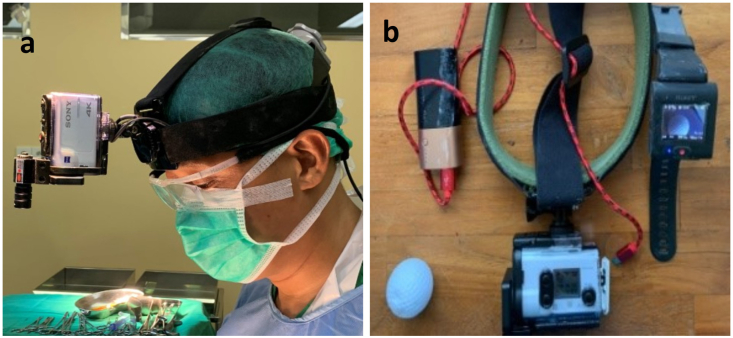
a) Physical appearance of the Sony FDR-X3000 when mounted; b) Physical appearance of the Sony FDR-X3000 when unmounted.

**Fig. 3:**
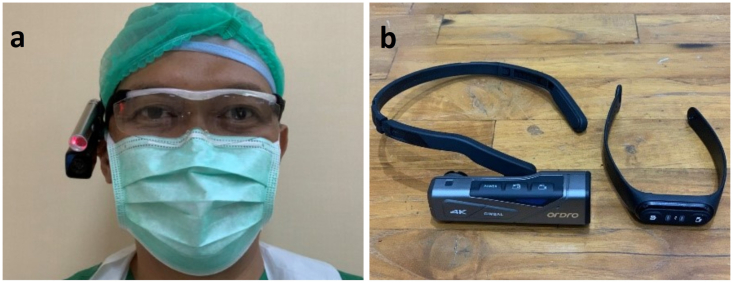
a) Physical appearance of the Ordro EP7 when mounted; b) Physical appearance of the Ordro EP7 when unmounted.

This study compared three types of cameras by assessing the camera settings before the operation and after surgery. Table 1 shows the three cameras’ specifications. Physical comparison of the cameras can be seen in Fig. 1, Fig. 2, Fig. 3 (see Table 2).

**Table 1:** Camera specifications.

Cameras	Panasonic HC-V770	Sony FDR-X3000	Ordro EP7
Resolution	Up to 1080P/120FPS	4608 × 2592	1080/30FPS
Video Format	AVCDH MP4	MPEG-4, XAVC S, H264	MOV H.264
Sensor	1/2.3 type (1/2.3″) BSI MOS Sensor	BSI CMOS	Sony High-sensitivity CMOS
Image Stabilizer	HYBRID O.I.S. with Active Mode, O.I.S. Lock, Level Shot Function	Optical/Optically Stabilized Ultra-Wide Lens	
Pixel	12 MP	8 MP	8 MP
Optical zoom	20×	–	–
Build-in Wi-Fi	Yes	Yes	Yes
Build-in Bluetooth	Yes	Yes	Yes
Microphone	5.1ch Surround, Zoom, Focus and Stereo Microphone	Built-in Stereo Mic	Built-in MIC
USB	USB 2.0 High Speed	USB 2.0	Micro-USB
Working Voltage	5.0 V	5.0 V	4.2 V
Power	Rechargeable Built-in Lithium-Ion	Rechargeable Built-in Lithium-Ion 1.89 A Battery	Rechargeable Built-in Lithium-Ion 850mAh Battery
Working current	1940mAh	800mAh	300mAh
External memory card	SD + Internal memory	Micro SD + Memory Stick Duo	Micro SD Support maximum 64 GB TF Card
Compatibility	Windows XP or above/Mac OSX 10.5 or above	Windows XP or above/Mac OSX 10.5 or above	Windows XP or above/Mac OSX 10.5 or above
Size	2.6″W*2.9″H *5.5″D	48*53*19 mm	97*27*26 mm
Weight	354 g	114 g	70 g
Price *USD	671.1	346.92	182.41
Physical appearance of the camera	(Fig. 1)	(Fig. 2)	(Fig. 3)

[[9], [10], [11]].

**Table 2:** Cameras comparison.

Cameras	Panasonic HC-V770	Sony FDR-X3000	Ordro EP7
**Time required for settings**	10–15 min	5–10 min	<5 min
**Comfort**			
**Short surgeries (<1 h)**	Comfort	Comfort	Comfort
**Medium surgeries (1–2 h)**	Comfort	Slight Discomfort	Comfort
**Long surgeries (> 2 h)**	Comfort	Discomfort	Slightly Discomfort
**Vantage point**	Fixed from top	Depict surgeon's eyes	Depict surgeon's eyes
**Video's angle**	Wide and narrow	Two options, Narrow and wide	Wide
**Focus on operation field**	Steady focus, often obstructed by operator head, vantage point not detailed	Focus, detailed on target	Focus, detailed on target
**Autofocus**	Yes	Yes	None
**Details on superficial organs (ex: abdominal wall and intestine)**	Good	Great	Fair
**Details on visceral organs (vascular, Biliary duct)**	Fair	Good	Poor
**Real time zoom**	Yes	None	None
**Zoom on editing**	Yes	Yes	Fair
**Surgeon's review**	Comfortable, due to fixed top mount	Easy adjustable head strap, heavy for head mounting, not suitable for long procedures, gave neck stiffness	Comfortable and light for head mounting, could be used for any duration surgery
**Time required for dismounting**	10–15 min	5–10 min	<5 min
**Superficial procedure**	(Fig. 4a)	(Fig. 5a)	(Fig. 6a)
**Visceral procedure**	(Fig. 4b)	(Fig. 5b)	(Fig. 6b)

For the Panasonic HC-V770, we used an inverted L-shaped stand with the tip fixed on the operating table and the other end mounted with a flexible arm and camera mounting. We connected it with a power bank with an extended cable to anticipate if the surgery took more than 2 h. Our Sony FDR-X3000 and Ordro EP7 action camera were mounted on the operator's head and connected to the power bank for medium and long surgery. We modified the head mounting for Sony FDR-X3000 by adding a clip to the inner shell of the construction helmet that was integrated with the head mounting to give more stability to the cameras. Ordro EP7 action camera was ready to use with a headband that was provided in the retail package.

We recorded 30 surgeries, from 10 patients for each camera in total for 5 superficial and 5 visceral surgery procedures. The Institutional Review Board of Faculty of Medicine, Public Health and Nursing approved this study (KE/FK/0796/EC/2018).

## 3 Results

**Fig. 4:**
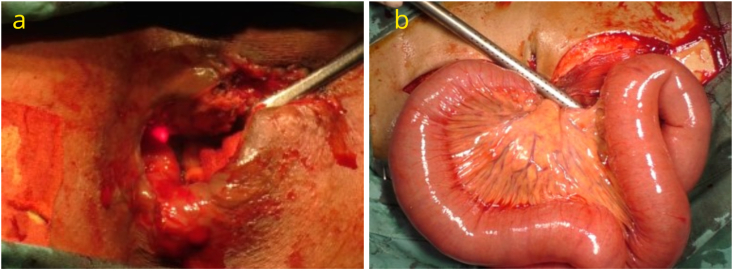
a) Picture quality of Panasonic HC-V770 during perianal fistula surgery; b) Picture quality of Panasonic HC-V770 during laparotomy surgery.

**Fig. 5:**
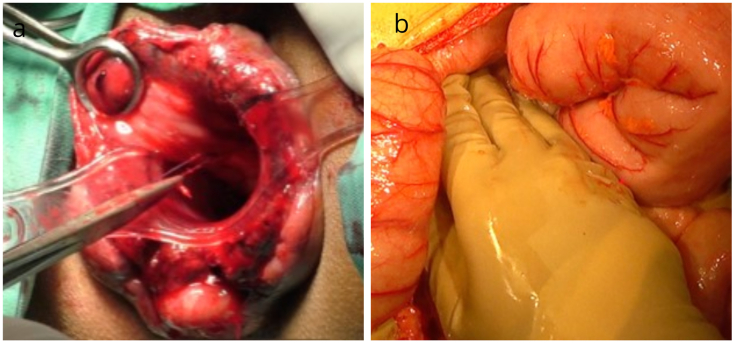
a) Picture quality of Sony FDR-X3000 during perianal haemorrhoid surgery; b) Picture quality of Sony FDR-X3000 during laparotomy surgery.

**Fig. 6:**
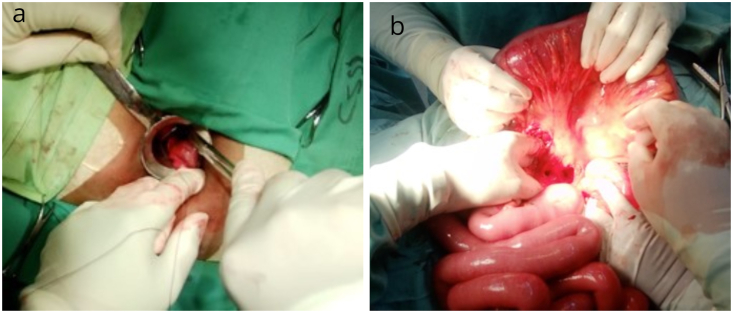
a) Picture quality of Ordro EP7 during perianal fistula surgery; b) Picture quality of Ordro EP7 during laparotomy surgery.

All patients who agreed to have their surgery recorded on our computer system

were offered the opportunity to see highlights of their surgery recorded simultaneously on the digital video recorder. The comparison of the three cameras is shown in the following table. Sample images of all cameras during superficial organ surgery can be seen in Fig. 4, Fig. 5, Fig. 6a. While the pictures during performing deep organ, surgery are seen in Fig. 4, Fig. 5, Fig. 6b.

## 4 Discussion

This study compared three types of cameras, namely Panasonic HC-V770 Full-HD Camcorder, Sony FDR X3000 Action Camera, and Ordro EP7 Hands-free Head-wearing Mini DV Camera. The most popular cameras in this market are GoPro Action Cameras (several models), Contour HD Helmet Camera, Panasonic HX-A100 POV Camcorder, and Google Glass. The ideal camera to record surgeries should be small, lightweight, comfortable, user friendly, able to depict surgeon's view, provide high definition images and videos, has long battery life, affordable, and enable an easy image or video management. To this date, this ideal kind of camera does not exist.

In this study, Panasonic HC-V770 Full-HD Camcorder needed 10–15 min for setting, Sony FDR X3000 took 5–10 min, and Ordro EP7 took less than 5 min. The Panasonic camcorder takes time for setting and dismounting, which made it more suitable for long surgeries, for example laparotomy, which do not need organ details and the procedures are done in superficial organ because it stands on external mounting. The camera being mounted on an external stand made it the most comfortable for the surgeon, providing steady vantage point and focus, with good image quality, that could be zoomed while recording, but may be obstructed by the surgeon's head at times.

Both action cameras need shorter time to set and dismount, able to depict surgeon's view, and could be controlled while recording using iOS or Android based Smartphone application. However, neither could be zoomed, and both had limited battery life (which could be solved by connecting common external power battery similar to phone's). Also, if initial camera direction setting was off, the entire recording would fail to point in the correct direction.

Sony FDR X3000 Action Camera has a high resolution sensor to give the best image quality among the three, even when the image was zoomed in the editing process, the fast autofocus, and the ability to be mounted of forehead with head band made it suitable for short to medium surgeries which require details, such as bile duct attachment and procedures on retroperitoneal or pelvic organs. However, its weight made it not suitable for long surgeries because neck stiffness occurred.

Ordro EP7 is a versatile head mounted action camera, lightweight and convenient to use and set. It is comfortable for any kinds of surgery, but the image quality was poor for visceral structures, even for superficial structures, hence zooming the image during editing was unfeasible, and the autofocus was slow. Setting the image quality to the best option may aid to slightly improve the output.

Previous study using head mounted Sony FDR-X3000R for liver transplant recording, very helpful for education supplementation due to high image quality and easy to edit. The most popular camera to record surgery procedures today is the GoPro Hero 6, due to high availability in the market and it is easy to use but has limited image quality [4,12]. Another study modified their own camera, that met the criteria for perfect surgical recording: narrow vantage point, HD image, detailed to small structure, head mounted, controlled from distance, can zoom, no need post record editing and the video can be used directly after recording, but it is expensive, heavy, less comfortable and not practical [13]. Technology's role in surgery is expected to continue to increase, with a projected $5.1 billion market for head mounted displays (HMDs) in the health care industry within the next decade [14]. In the end, whether cameras are good depends on what procedures we want to record, for example, the use of a combination of top mounted camcorder such as the Panasonic HC-V770 for fixed wide vantage point and head mounted action-cam for moving vantage points can be considered.

This study had limitations as we only used one particular camera for each camera type which does not rule the possibility of different result between cameras in the same type, we didn't use standardized surgical camera instead we use action camera although we need make a custom mounting for the camera and the comparation was made by one surgeon which does not rule the possibility of different experience between surgeons. Further study with larger sample size, more camera types, and more evaluator needed to determine the best camera for surgery recording.

## 5 Conclusions

The most important point of surgery recording is image quality. Panasonic HC-V770 Full-HD Camcorder and Sony FDR X3000 Action Camera have good image quality, and the Camcorder excelled in longer surgeries due to its comfort, whereas the Action Camera excelled in shorter surgeries due to its ease of use and settings. Ordro EP7 was the most comfortable among all but due to its lower image quality, it became the least favourite camera for surgery recordings.

## Declaration of competing interest

The authors declare that they have no competing interests.

## Registration of research studies

1.Registry used: Research Registry
2.Unique Identifying number or registration ID: researchregistry6193
3.Hyperlink to your specific registration: https://www.researchregistry.com/browse-the-registry#user-researchregistry/registerresearchdetails/5f9e0129dc4a190018589a28/

## Availability of data and material

Not applicable.
